# Chronotype and Psychiatric Disorders

**DOI:** 10.1007/s40675-018-0113-8

**Published:** 2018-04-16

**Authors:** Liia Kivelä, Marinos Rodolfos Papadopoulos, Niki Antypa

**Affiliations:** Department of Clinical Psychology, Institute of Psychology, Leiden University, Wassenaarseweg 52, 2333 AK Leiden, The Netherlands

**Keywords:** Chronotype, Eveningness, Depression, Seasonal affective disorder, Delayed sleep phase syndrome, Substance abuse, Eating disorder, Bipolar disorder, Anxiety disorder

## Abstract

**Purpose of Review:**

Chronotype, reflecting interindividual differences in daily activity patterns and sleep-wake cycles, is intrinsically connected with well-being. Research indicates increased risk of many adverse mental health outcomes for evening-type individuals. Here, we provide an overview of the current evidence available on the relationship between chronotype and psychiatric disorders.

**Recent Findings:**

The association between eveningness and depression is well established cross-sectionally, with preliminary support from longitudinal studies. The mechanisms underlying this relationship warrant further research; deficient cognitive-emotional processes have recently been implicated. Eveningness is associated with unhealthy lifestyle habits, and the propensity of evening types to addiction has been recognized. Chronotype may also be implicated in disordered eating.

**Summary:**

Eveningness is associated with depression—including seasonal affective disorder (SAD)—and substance dependence, while support for a relation with anxiety disorders and psychosis is lacking. In bipolar disorder, chronotype is linked to depression but not mania. Eveningness is also related to sleep disturbances and poor lifestyle habits, which may increase risk for psychiatric disorders.

## Introduction

Circadian rhythmicity in humans is apparent in a wide range of biological and behavioral functions, from hormone secretion and body temperature to sleep-wake patterns and socialization. These endogenous diurnal variations are determined by time “chrono,” and controlled by our internal circadian clocks and external environmental cues “zeitgebers,” such as light [1, 2].

Circadian expression varies among people and is mainly influenced by hereditary and environmental influences, age, and sex **[**1, 2]. Although considered to lie on a continuum from morningness to eveningness, time-of-day preference is often characterized with the concept of chronotype. This classification comprises of three circadian types: the morning, the intermediate and the evening type. Morning-type individuals show a preference for going to bed and waking up early, and accomplish the peak of their mental and physical performance in the early part of the day. Evening types, in contrast, achieve their peak towards the end of the day and consequently prefer later bed and wake up times. Around 40% of adults belong in one of the two extreme circadian types, while most people fall along the middle part of the continuum [2]. Chronotype has traditionally been measured with the Morningness-Eveningness Questionnaire [3] and more recently with the Munich Chronotype Questionnaire [4], while other scales have also been developed, especially in shorter forms [2].

The enthusiasm for the investigation of chronotypes has grown quickly in the last 20 years, and it is now acknowledged that circadian preference can influence physical and mental health, in terms of well-being, but also sickness and disease [5•]. Indeed, the increased prevalence of evening types among patients with mood disorders, substance abuse, and sleep disturbances has been recently established [5•, 6–8]. The aim of this review is to provide an overview of psychiatric disorders and their associations with the morningness-eveningness dimension.

## Chronotype and Mood Disorders

### Chronotype and Depression in Young Populations

Eveningness has previously been associated with depressive symptoms in university students [9–11]. More recently, studies have demonstrated increased risk for children and adolescents as well. In two samples of Taiwanese school children, evening types were more likely than morning types to report both internalizing problems—including depression and somatic complaints—and suicidality [12, 13•]. Eveningness was also associated with depression independent of insomnia in Australian high school students [14]. In another recent study of 255 children and adolescents aged 11 to 19 years, evening-type individuals were more likely to report a past diagnosis of a depressive disorder, and an earlier onset of depressive symptoms [15••]. Further, eveningness prospectively predicted both increases in depressive symptoms and onset of a depressive episode over a 12-month period, even when prior depression was controlled for [15••]. A delayed dim light melatonin onset (DLMO) has also been associated with increased negative affect and reduced positive affect in young adolescents [16•]. Hence, eveningness appears to be an independent risk factor for both subclinical depressive symptoms and onset of a depressive disorder among young people, though more studies are warranted to confirm this observation.

### Chronotype and Depression in Adult and Clinical Populations

Among adults, eveningness has been associated with an increased likelihood of reporting depressive symptomatology, increased symptom severity, and being diagnosed with a depressive disorder [5•, 17–20, 21•, 22]. Further, these findings have been replicated in large-scale studies cross-culturally. In a sample of 10,503 Finnish adults, eveningness was associated with increased odds of reporting depressive symptoms, having a current diagnosis of a depressive disorder, and taking antidepressant medication [21•]. The association between the evening chronotype and major depressive disorder (MDD) was replicated in another recent study utilizing a large Dutch cohort [22]. Among Korean young adults, eveningness has been linked to a “depressive temperament,” a subclinical manifestation of depression [23].

Some debate exists over whether depressed individuals are more likely to be evening types as compared to their non-depressed counterparts, a position supported by some [10, 13•, 24•]. However, others have found most depressed individuals to be intermediate types [25, 26•, 27]. It has been argued that rather than evening types being overrepresented in depressed populations, morning types tend to be underrepresented [27]. In a rather contradictory finding, in a study involving psychiatric inpatients, morning types were more likely to suffer from depression [28].

Among depressed individuals, evening types tend to exhibit worse symptom severity and report increased psychological distress, higher suicidality and more impairment in their daily lives (including work), and more symptoms of anxiety [24•, 25, 26•, 29]. However, eveningness does not appear to be associated with either suicidal ideation or attempt among individuals without a psychiatric diagnosis [11]. Eveningness conveys additional risk through its association with non-remission of depression [26•]. Further, evening preference is associated with increased comorbidity in clinical samples [30].

The underlying mechanisms linking eveningness and depression have been explored recently. Eveningness has been associated with a lower behavioral activation system (BAS), which in turn leads to lower reward responsiveness and lower positive affect, and consequently depressive symptoms [18]. Some have reported that depressive symptoms tend to worsen towards the evening hours, possibly explaining why evening-type people might spend more time in depressed mood states [26•]. However, we have shown that evening-type individuals experience worse mood in the mornings [22]. The timing of reduced positive affect or mood worsening in evening types requires further research. It has been argued that evening types also exhibit difficulties with affect regulation, consequently putting them at risk for experiencing affective disturbances, such as depression [18]. Indeed, evening types have been shown to have impaired emotion regulation [31•], especially higher self-blame and reduced positive reappraisal [32]. We also found increased rumination levels in evening types, which in turn explained the link between eveningness and depression [33•]. Sleep disturbances have also been proposed as mediators, wherein the disturbed or insufficient sleep of evening-type individuals places them at risk for depression [2, 32]. However, in a sample of 1170 Japanese adults, eveningness was associated with an increased incidence of depressive states independent of sleep complaints, such as poor subjective sleep quality and daytime sleepiness [19]. Finally, we recently found that social jetlag, the misalignment between the internal clock and the social clock (i.e., timing of social obligations) was related neither to depression, nor to depressive symptoms [34]. Longitudinal studies are needed for establishing mediators of the eveningness—depression relationship.

### Chronotype and Bipolar Disorder

Evidence for an association between eveningness and bipolar disorder has been reported; bipolar patients are more likely to be evening types [8, 24•, 35, 36], and this finding has been replicated using both objective (actigraphy) and subjective sleep measures [37••]. Further, although there were no differences in circadian preference when bipolar I and bipolar II patients were compared, eveningness was associated with elevated depressive symptoms and the use of mood stabilizers among all patients [35]. Evening preference is also associated with depression proneness, a vulnerability trait for bipolar disorder [38]. Null findings have also been found; in a sample of 1468 psychiatric inpatients, bipolar disorder was not specifically associated with any chronotype [28]. Overall, eveningness appears to be more directly associated with symptoms of depression rather than mania among bipolar patients [8].

### Chronotype and Seasonal Affective Disorder

Evening-type adolescents have been found to report higher mood seasonality than morning and intermediate types [39]. Further, in a 3-year prospective study with a community sample of 244 adults, seasonal worsening of mood during winter months was accompanied with a shift towards eveningness [40]. Individuals with seasonal affective disorder (SAD) are also more likely to have an evening preference throughout the year [41, 42]. An association between delayed sleep phase syndrome (DSPS), a disturbance related to extreme eveningness, and SAD has also been reported [42]. Positive results have been obtained with light therapy for patients with both seasonal and non-seasonal depression, although it is unclear whether the treatment effects are caused by circadian phase advances (i.e., a shift towards morningness) [43, 44•].

## Chronotype and Anxiety Disorders

### Chronotype and Anxiety Disorders in Young Populations

Findings on the relationship between chronotype and anxiety in adolescents and young adults are contradictory. Eveningness has been associated with trait anxiety in female adolescents [45]. A greater proportion of evening types has been observed among adolescents with high anxiety, while morning preference was more prevalent among those with low anxiety [46]. Increased anxiety and psychosomatic symptoms have also been observed among evening-type undergraduate students [10, 47]. Among 142 medical students, evening-type students reported experiencing more anxiety in general, and more obsessive-compulsive and phobic symptoms in particular, than their intermediate and morning counterparts [48]. However, others have found no association between chronotype and either state or trait anxiety in young adults [49–52]. Eveningness was also not associated with anxiety disorders (obsessive-compulsive disorder, panic disorder, separation anxiety, social phobia) among adolescents after sleep complaints were controlled for [14].

### Chronotype and Anxiety Disorders in Adults

In adult samples, evening types appear to be more vulnerable to anxiety [6, 23, 24•, 28]. Among Korean adults, eveningness has been associated with an anxious temperament [23]. Evening types have also been shown to exhibit increased physiological arousal, as indicated by increased heart rate and systolic blood pressure and decreased heart rate variability, both during conditions of rest and stress [53]. Irregular rhythms of melatonin release in students have also been associated with psychosomatic complaints, including anxiety [54]. Indeed, patients with anxiety disorders exhibit greater evening preference than those without such diagnosis [24•, 28]. However, anxiety disorders (social phobia, panic disorder with/without agoraphobia, generalized anxiety disorder) were not related to eveningness in a large cohort study [22]. Similarly, in 1548 patients with fibromyalgia, no differences were found between chronotypes on anxiety symptoms [55]. However, more consistent findings have been recently reported with regard to post-traumatic stress disorder (PTSD), with evening-type firefighters [56] and military veterans [57] showing increased PTSD symptoms compared to other chronotypes.

## Chronotype and Psychotic Disorders

The association between chronotype and psychotic disorders has so far received limited attention in comparison to other mental health conditions, and current findings do not point to a straightforward link between circadian preference and psychosis. In a large sample of psychiatric inpatients, psychotic patients were more frequently morning-oriented [28]. However, in another study of 496 participants aged 12 to 30 years, psychotic patients exhibited higher levels of eveningness than their non-psychiatric comparisons [24•]. Still, others have found no association between chronotype and psychosis among either psychotic patients or those at risk of such disorders [58, 59].

## Chronotype and Addictive Disorders

### Chronotype and Addictive Disorders in Young Populations

The evening chronotype is considered a major risk factor for substance use among young people [60–62, 63•]. Adolescents and young adults with a tendency towards eveningness report higher levels of both experimental and habitual cigarette smoking as compared to their morning-oriented peers [12, 50, 60, 62, 64–68]. More alcohol is also used by evening-type adolescents [12, 64, 68], and excessive alcohol consumption among college students is associated with the late chronotype [60, 62, 66, 67]. Among students, evening types report higher consumption of illegal drugs—including cannabis, cocaine, ecstasy, and amphetamines—as compared to morning and intermediate types [60, 63•, 66]. In a sample of 2410 Ethiopian college students, an association was found between the use of the drug khat, a herbal stimulant, and evening orientation, indicating that the association between eveningness and substance use can be found in non-Western cultures too [62].

The evening chronotype has also recently been associated with compulsive internet use among young adults [47]. Others have shown evening-type individuals to exhibit increased engagement with computer games and social media, and problematic mobile phone usage [69–71]. Evening types also use more electronic media at bedtime [72].

### Chronotype and Addictive Disorders in Adults

In adult samples, evening types are more likely to smoke cigarettes and to consume alcohol, also in higher amounts [21•, 22, 60, 65, 73–75]. Indeed, evening types are more than twice as likely to be smokers than intermediate types [76]. Evening types are also at greater risk of developing alcohol dependence [50, 60, 61, 67]. It has been proposed that evening-type individuals, who experience higher arousal in late hours, use alcohol in the evenings for its sedative properties [60]. Further, a recent study in 333 adult substance abuse outpatients found that the evening chronotype was associated with cannabis addiction, non-substance addiction (such as gambling), and poly-substance use, although no association with addiction severity was found [77•]. In accordance, individuals diagnosed with an addictive disorder are more likely to be evening type [28].

Neuroimaging studies have investigated how circadian alterations could influence reward-related brain activity and in turn predispose individuals to substance abuse [61]. In an fMRI paradigm, when the neural activity of 13 morning and 21 evening-type participants was compared in response to a monetary reward, reductions in medial prefrontal cortex (mPFC) activity were observed among evening-type individuals when anticipating the reward. Evening types also exhibited increased ventral striatum reactivity in response to receiving the reward. These alterations were further correlated with greater reported alcohol consumption and more symptoms of alcohol dependence, indicating that altered reward processing may explain the greater propensity of evening-type individuals to substance use [61]. Eveningness has also been associated with a personality type characterized by greater impulsivity, disinhibition, and sensation seeking, which may in turn increase the tendency of these individuals towards engaging in unhealthy behaviors [10, 78, 79].

## Chronotype and Sleep Disorders

### Chronotype and Sleep Disorders in Adolescents

The evening chronotype is more prevalent in adolescence and early adulthood as compared to childhood and later adulthood [2]. This shift towards eveningness starts at roughly 13 years of age and peaks at age 20 [80–83]. Biological, social, and behavioral influences, such as pubertal changes, lessened parental control, and recreational activities, are thought to predispose teenagers towards eveningness [80, 83]. However, while chronotypes vary both between and within individuals, social schedules are not always adaptable, which may subject evening-type adolescents to chronic sleep deprivation [83–85]. Indeed, evening-type adolescents report more irregular sleep-wake cycles, poorer subjective sleep quality, and more daytime sleepiness, also resulting in more napping [64, 80, 82]. Delayed sleep phase syndrome (DSPS; characterized by a frequently delayed sleep-wake cycle) has also been associated with eveningness in adolescence, and may result in chronic fatigue, low mood, and academic difficulties, when individuals are unable to synchronize their internal schedules with external (e.g., school) requirements [85–87]. Consequently, the need to adapt work and school schedules to chronotype has been brought forward [65].

### Chronotype and Sleep Disorders in Adults

Common sleep complaints reported by evening-type adults include decreased subjective sleep quality, insufficient sleep, excessive daytime sleepiness, and trouble initiating sleep [19, 26•, 66, 74, 75, 88–90]. Evening types also report more severe insomnia symptoms [26•, 89, 90]. However, in some samples, insomnia has been found to be more prevalent among morning types [22]. Hypersomniacs may more frequently be evening types [91]. Evening-type adults also experience more frequent nightmares and use more hypnotic medications as compared to morning and intermediate types [26•, 51, 89, 92]. However, sleep apnea has been associated with both the morning and evening chronotypes among overweight and obese individuals [93, 94]. Evening types similarly exhibit a heightened risk for the development of DSPS and demonstrate a sleep pattern that is 2–3 h later than that of morning types, as indicated by delayed changes in core body temperature and melatonin production during typical sleep hours [95, 96]. In accordance, most DSPS patients are evening types [96]. Eveningness with comorbid sleep complaints significantly increases the risk for serious mental health problems [20, 26•, 90, 97•].

## Chronotype and Eating Behavior/Disorders

Morning-type individuals tend to exhibit healthier and more regular eating habits and higher control over their eating as compared to evening types [74, 88, 98, 99]. Personality differences may help explain the aforementioned findings, whereby neuroticism might predispose individuals to both eveningness and uncontrolled eating [99]. Evening types also tend to be less physically active and report poorer perceived health in general, as compared to morning and intermediate types [100•]. However, although some have reported eveningness to be associated with an increased body mass index (BMI) [88, 101], chronotype does not appear to be a risk factor for obesity [45, 74, 75, 98, 99, 100•, 102]. In two large cohorts, evening types were even found to have lower body weight than other chronotypes [21•, 22].

Evidence for an association between chronotype and specific eating disorders is limited. An association between bulimic behavior and evening preference has been demonstrated in university students [103]. Individuals with binge eating behaviors have been found to be more likely to be evening types [102]. Lower morning alertness has also been linked to higher emotional eating [104], and eveningness was associated with less dietary restrain and more uncontrolled eating among students [98]. Eveningness was more prevalent among eating disorder patients as compared to healthy controls, and in 46 patients followed prospectively, reductions in eating disorder symptoms were also associated with a shift towards morningness [105]. However, in another study in psychiatric inpatients, no association was found between eating disorder diagnosis and circadian type [28].

## Conclusions

A considerable body of evidence posits an increased risk of a number of adverse mental health outcomes for individuals who exhibit a preference for evening hours, as compared to those who are naturally inclined to start their days earlier. A summary of the reviewed evidence can be found in Table 1.

**Table 1:** Summary of the findings of chronotype as a risk factor for psychiatric disorders

Diagnosis	Chronotype	Population	Comments
Depression (MDD)	Evening	• Children/adolescents • Adults	• ↑ Prevalence • Earlier onset of symptoms • ↑ Mood seasonality • ↑ Prevalence (incl. SAD) • ↑ Antidepressant use • ↑ Symptom severity (incl. suicidality) • ↑ Chronicity and comorbidity
Bipolar disorder	Evening		• Vulnerability to depression • No clear link with mania • ↑ Mood stabilizer use
Anxiety disorders	Evening	• Adolescents • Adults	• Some subclinical symptoms (incl. psychosomatic) • Mixed findings (no association) • ↑ PTSD symptoms
Psychotic disorders			• No clear link with psychosis
Addictive disorders	Evening	• Adolescents • Adults	• ↑ Substance use (nicotine, alcohol, drugs) • ↑ Compulsive internet use • ↑ Substance use (nicotine, alcohol, drugs), gambling • ↑ Alcohol dependence • ↑ Addictive disorders
Sleep disorders	Evening	• Adolescents • Adults	• ↑ DSPS • ↑ Hypersomnia • ↑ Nightmares • ↑ Hypnotic medication use • Mixed findings for insomnia
Eating disorders	Evening		• ↑ Bulimic, binge-eating behaviors

*MDD* major depressive disorder, *SAD* seasonal affective disorder, *PTSD* post-traumatic stress disorder, *DSPS* delayed sleep phase syndrome

**Fig. 1:**
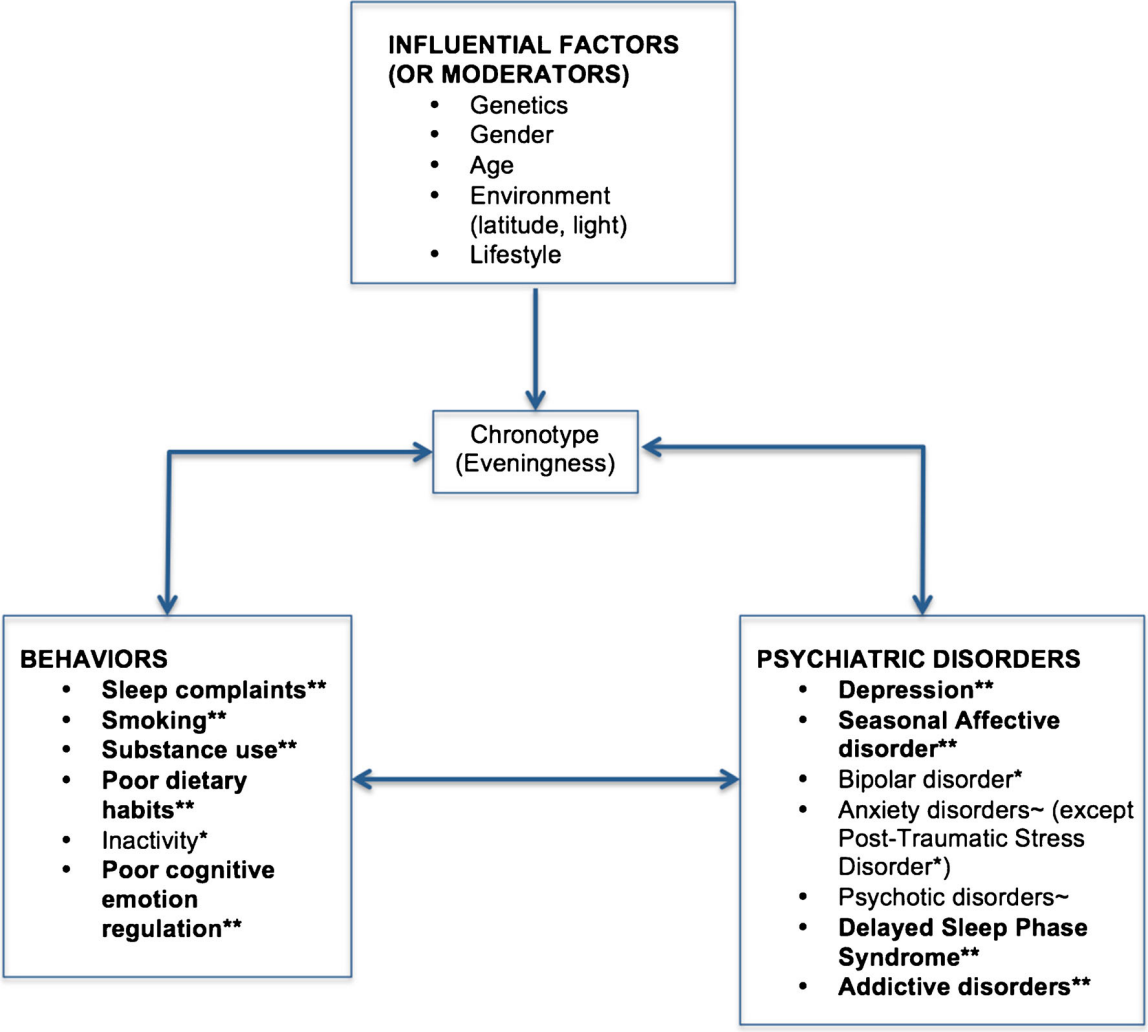
Associations between chronotype (eveningness), behavioral disturbances and psychiatric disorders, with **strong evidence (more than two studies showing an association), *weak evidence (1 or two studies show an association), and ~ mixed findings (some studies show an association whereas others do not)

Eveningness is related to affective disorders and especially depression; these include worse symptom severity, poorer prognosis, and higher seasonal mood variation among evening types. While these associations have been widely reported in cross-sectional studies, two recent longitudinal accounts have supported the notion that chronotype may be a risk factor for subsequent depression [15••, 32]. More prospective studies are needed in order to determine the predisposing role of eveningness, and how disease course may in turn influence chronotype. The associations between eveningness and depression are likely to include complex, bidirectional influences [7], and the direction of causality remains to be further established. Strengthening argumentation for causality also requires more research on the mechanisms underlying this relationship; while sleep disturbances have been proposed to explain the relationship, eveningness has been linked to depression independent of insomnia and other sleep complaints [14, 19, 26•]. Lately, studies have explored disturbed cognitive-emotional processes among evening types as a possible explanatory mechanism [31•, 32, 33•], and these findings warrant further replication. Evening types also show greater propensity for engaging in health-impairing behaviors—including substance use and poor dietary habits—and are represented at higher numbers among those with alcohol dependence and other addictive disorders. These associations are likely to further contribute to the increased prevalence of depression and other mental disorders among evening types (Fig. 1). Evening types have also been shown to exhibit some bulimic and binge-eating behaviors, but eating disorders should be further investigated in relation to chronotype.

Some methodological issues can be discussed. Most studies reviewed relied on retrospective self-report of both psychopathology and morning-evening preference, although some employed objective circadian measures such as actigraphy and body temperature [16•, 37••, 54, 95]. However, these studies were also those with the smallest sample sizes. Furthermore, despite the consistent evidence linking eveningness with psychiatric outcomes (especially depression), effect sizes of these associations vary dramatically, from large to small [19, 20, 21•, 22]. A recent meta-analysis on the association between chronotype and depressive symptoms included 36 studies (*n* = 15,734) and showed a small overall effect size (*z* = − .20; 95% CI − .18 to − .23), with no evidence of publication bias, but some variability related to instrument measurement [7]. It is also noteworthy that the majority of studies have relied on non-clinical community or student samples, which may not generalize to other age groups or clinical populations. However, studies in clinical samples [25, 26•, 27–29, 44•, 77•, 86, 96] and those comparing psychiatric patients and healthy controls [22, 24•, 35, 36, 37••, 42, 58, 91, 105] have largely supported these findings, although confounders such as medication use may contribute to these group differences.

Although the outlined evidence paints a gloomy picture for evening types, it should be emphasized that eveningness alone is not likely to cause depression or other psychiatric disorders, and additional influences are likely to play a role. Research into the mechanisms underlying the chronotype-disorder link are also likely to provide targets for interventions that may help decrease the incidence of psychiatric disturbances among evening types; these might include sleep education, dietary advice, and cognitive-behavioral techniques. Such preventive efforts may be particularly relevant for adolescents and young adults who are more likely to be evening types, and especially since many mental disorders commonly first present during this developmental period. In conclusion, clinicians should be mindful that circadian preference may predispose individuals to psychopathology and influence disease course and treatment outcome among those already affected.
